# Supplementary material for: Comprehensive Study of Some Cyanobacteria in Moscow Waterbodies (Russia), Including Characteristics of the Toxigenic Microcystis aeruginosa Strains
Source: Toxins (Basel). 2025 Oct 14;17(10):506. doi: 10.3390/toxins17100506 (PMC12568284; doi:10.3390/toxins17100506)
Supplement: Supplementary file 1 [file toxins-17-00506-s001.zip › S2_Figures S1_S4.pdf]

Supplementary Materials: Comprehensive study of some cyanobacteria in Moscow waterbodies (Russia), including characteristics of the toxigenic *Microcystis aeruginosa* strains

Elena Kezlya, Elina Mironova, Ekaterina Chernova, Maria Gololobova, Andrei Mironov, Ekaterina Voya-kina, Yevhen Maltsev, Dina Snarskaya and Maxim Kulikovskiy

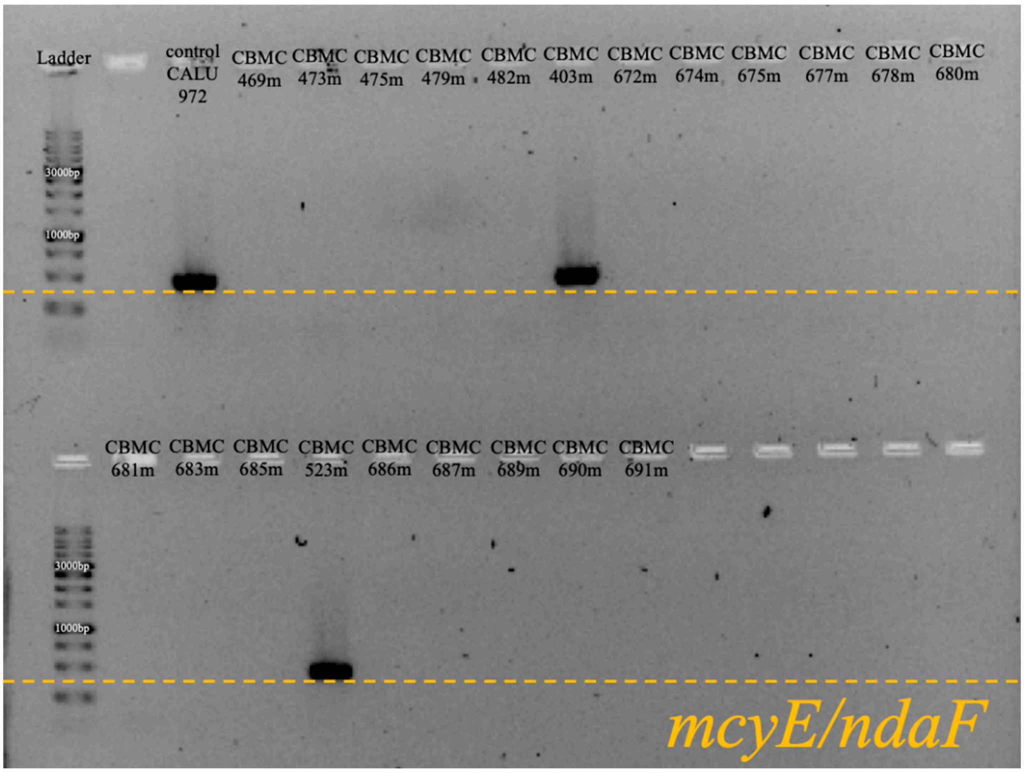

Figure S1. Electrophoresis gel photos of *mcy*E/ *nda*F biosynthesis gene amplification with primers HEPF/HEPR [46].

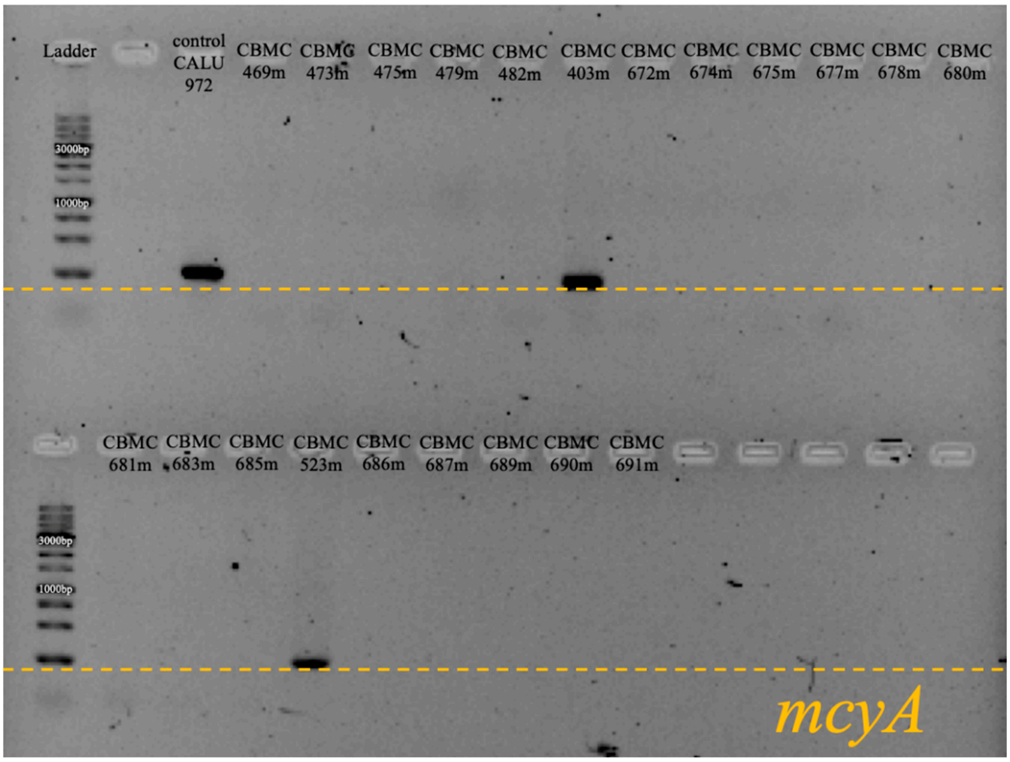

Figure S2. Electrophoresis gel photos of *mcy*A biosynthesis gene amplification with primers mcyACdF/mcyACdR [47].

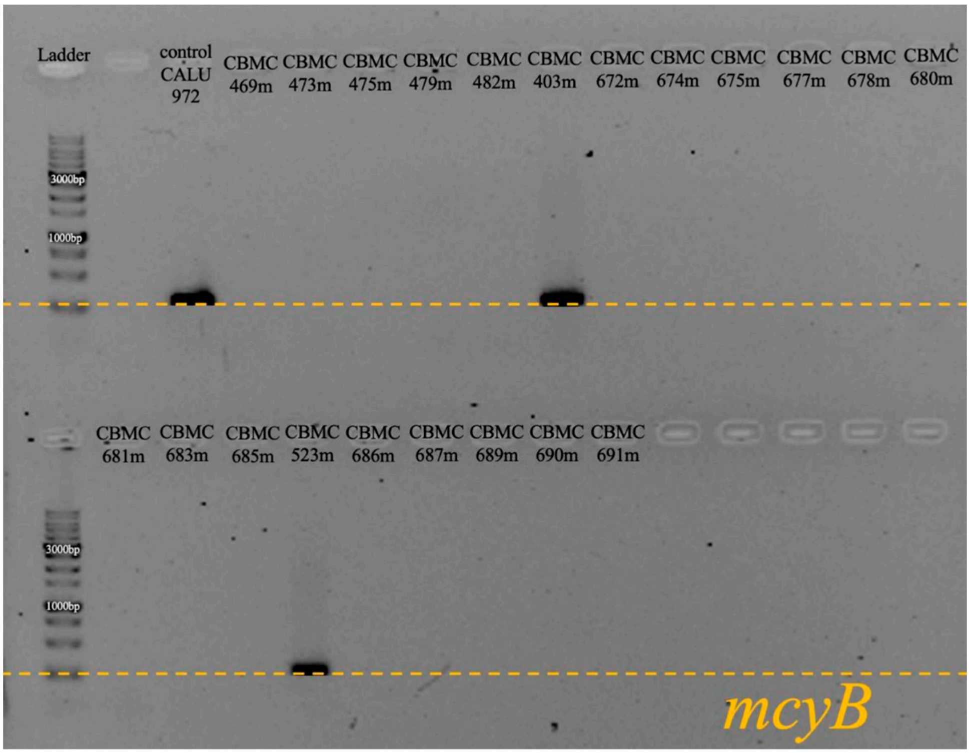

Figure S3. Electrophoresis gel photos of *mcy*B biosynthesis gene amplification with primers McyB-F/McyB-R [48].

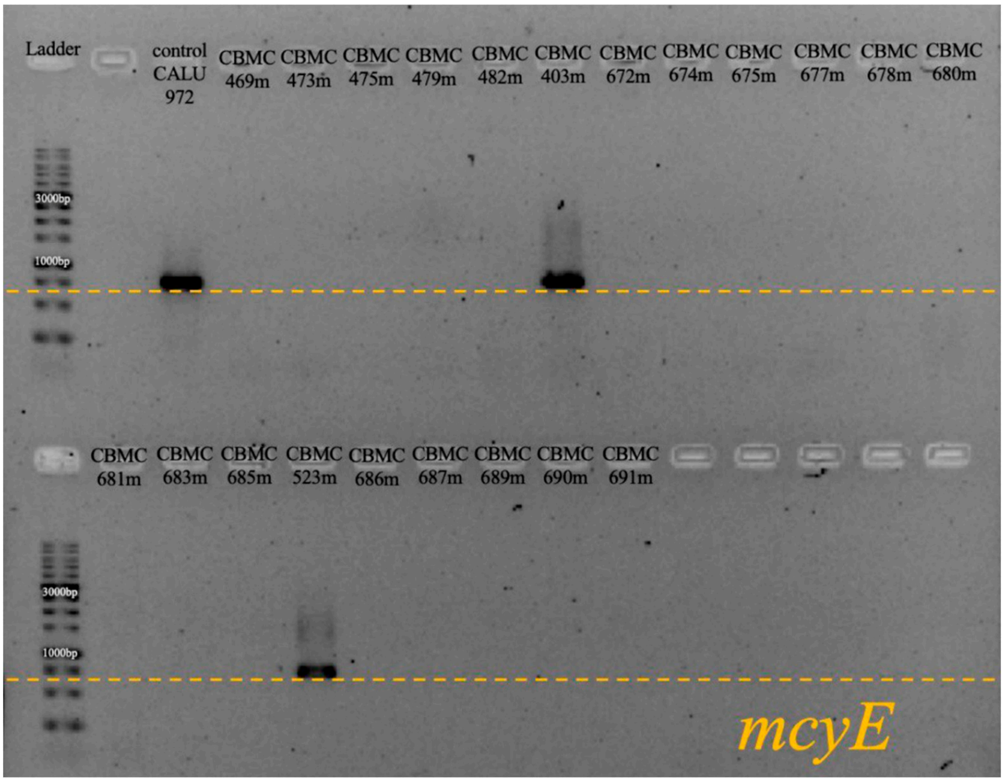

Figure S4. Electrophoresis gel photos of *mcy*B biosynthesis gene amplification with primers mcyE-F2/mcyE-R4 [49].
