# Supplementary material for: Comprehensive Study of Some Cyanobacteria in Moscow Waterbodies (Russia), Including Characteristics of the Toxigenic Microcystis aeruginosa Strains
Source: Toxins (Basel). 2025 Oct 14;17(10):506. doi: 10.3390/toxins17100506 (PMC12568284; doi:10.3390/toxins17100506)
Supplement: Supplementary file 1 [file toxins-17-00506-s001.zip › S3_Figures S5_S8.pdf]

Supplementary Materials: Comprehensive study of some cyanobacteria in Moscow waterbodies (Russia), including characteristics of the toxigenic *Microcystis aeruginosa* strains

Elena Kezlya, Elina Mironova, Ekaterina Chernova, Maria Gololobova, Andrei Mironov, Ekaterina Voya-kina, Yevhen Maltsev, Dina Snarskaya and Maxim Kulikovskiy

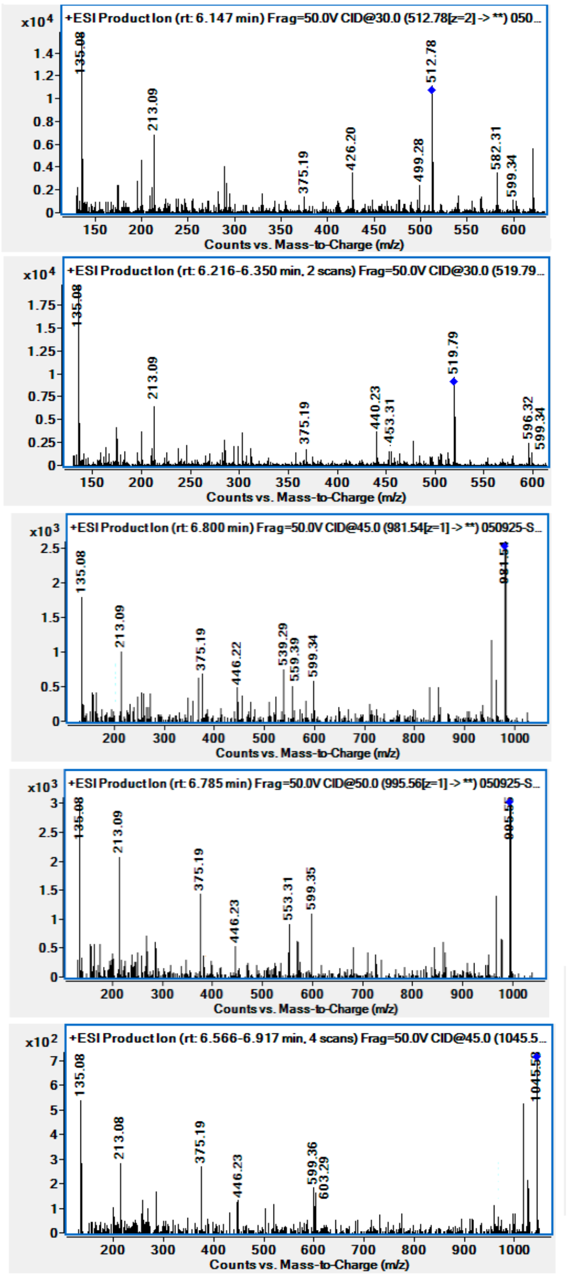

Supplementary Figure S5. Fragment spectra for MC congeners from standard solution. The precursor ions (from top to bottom): m/z 512.7829 ([M+2H]$^{2+}$ [D-Asp^3]MC-RR; m/z 519.79077 ([M+2H]$^{2+}$ MC-RR; m/z 981.54095 ([M+H]$^+$ [D-Asp^3]MC-LR); m/z 995.55658 ([M+H]$^+$ MC-LR); m/z 1045.53589 ([M+H]$^+$ MC-YR))

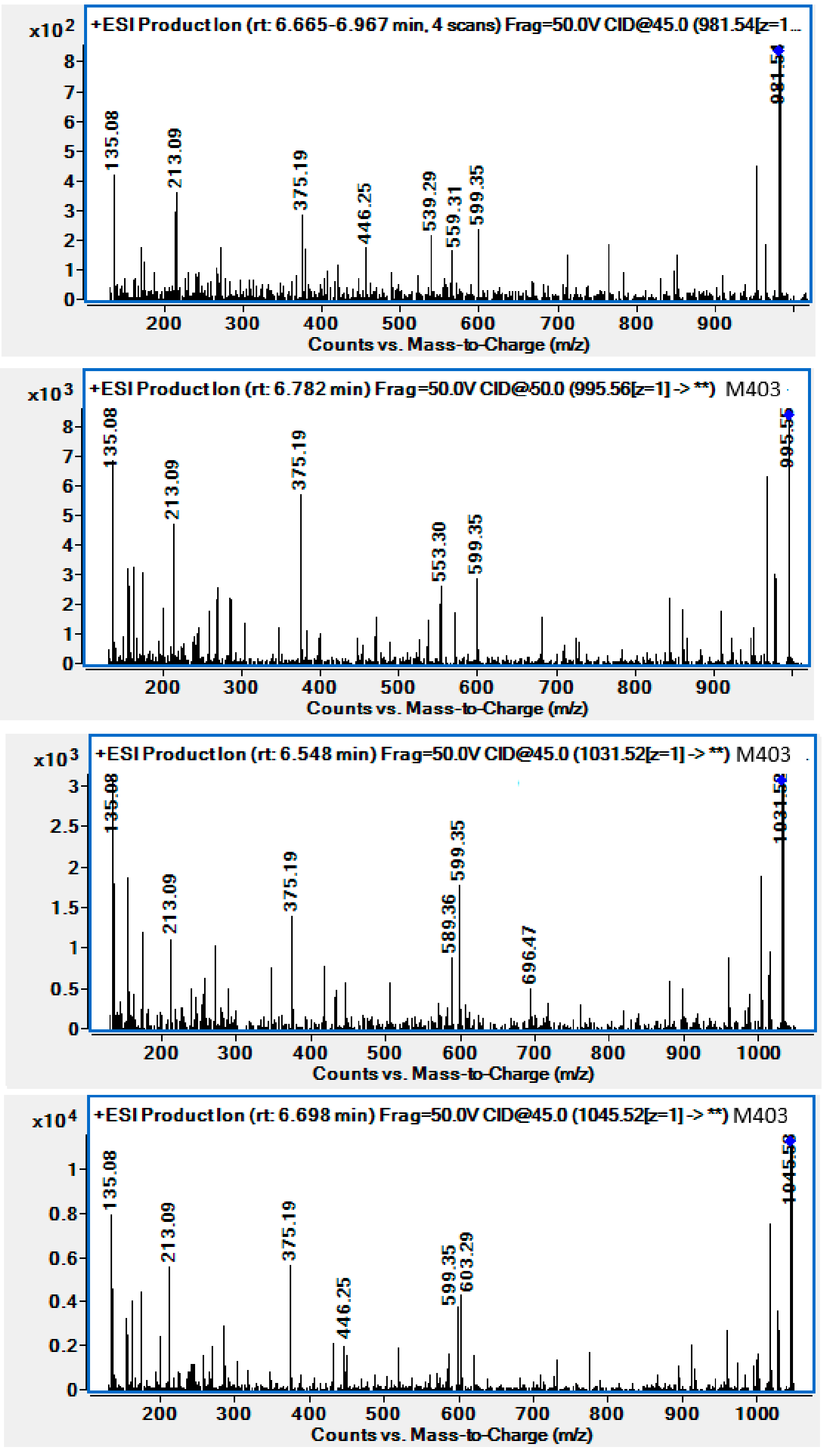

Supplementary Figure S6. Fragment spectra for MC congeners identified in biomass sample CBMC403m. The precursor ions (from top to bottom): m/z 981.54095 ([M+H]$^+$ [D-Asp^3]MC-LR); m/z 995.55658 ([M+H]$^+$ MC-LR); m/z 1031,5203 ([M+H]$^+$ [D-Asp^3]MC-YR); m/z 1045.53589 ([M+H]$^+$ MC-YR)).

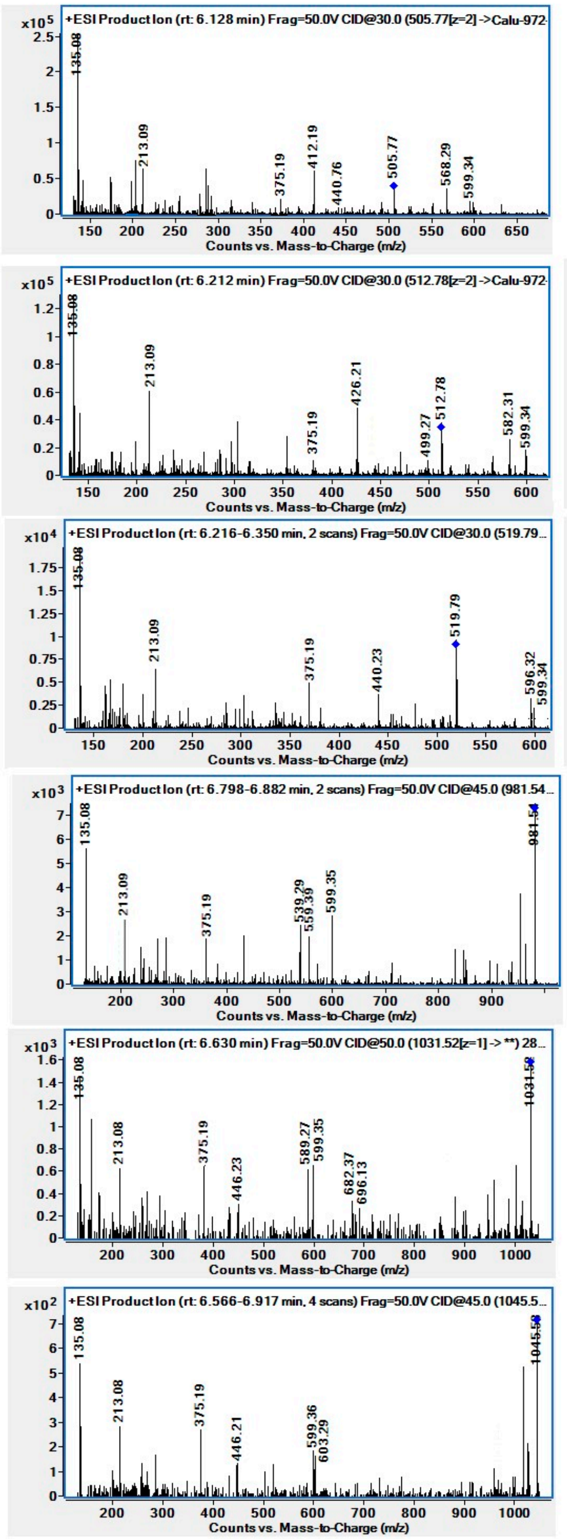

Supplementary Figure S7. Fragment spectra for MC congeners identified in biomass sample CALU972. The precursor ions (from top to bottom): m/z 505,7734 ($[M+2H]^{2+}$ [D-Asp^3, Dhb^7]]MC-RR; m/z 512.7829 ($[M+2H]^{2+}$ [D-Asp^3]MC-RR; m/z 519.79077 ($[M+2H]^{2+}$ MC-RR; m/z 981.54095 ($[M+H]^+$ [D-Asp^3]MC-LR); m/z 1031,5203 ($[M+H]^+$ [D-Asp^3]MC-YR); m/z 1045.53589 ($[M+H]^+$ MC-YR))

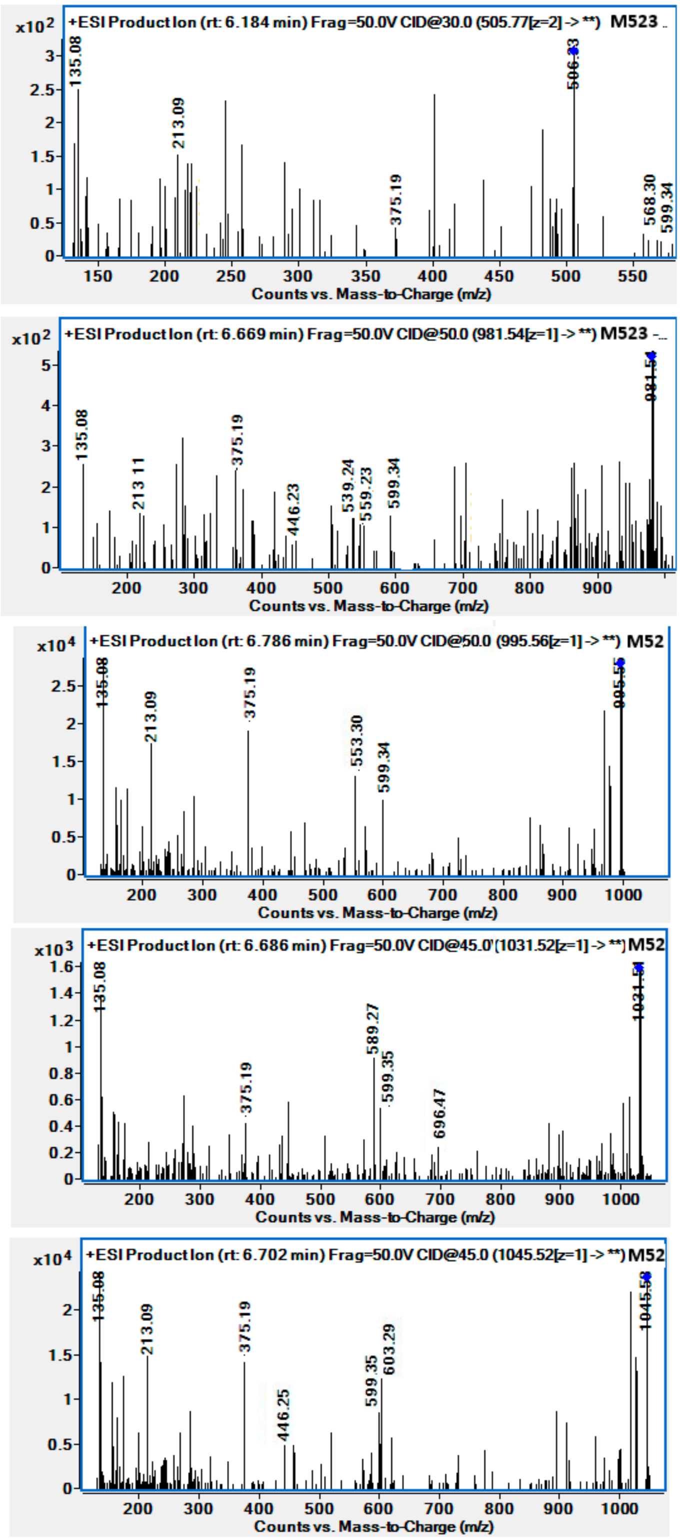

Supplementary Figure S8. Fragment spectra for MC congeners identified in biomass sample CBMC523m. The precursor ions (from top to bottom): m/z 505,7734 ($[M+2H]^{2+}$ [D-Asp^3, Dhb^7]]MC-RR; m/z 981.54095 ($[M+H]^+$ [D-Asp^3]MC-LR); m/z 995.55658 ($[M+H]^+$ MC-LR); m/z 1031,5203 ($[M+H]^+$ [D-Asp^3]MC-YR); m/z 1045.53589 ($[M+H]^+$ MC-YR))
